# Identification of a First Human Norovirus CD8^+^ T Cell Epitope Restricted to HLA-A^*^0201 Allele

**DOI:** 10.3389/fimmu.2018.02782

**Published:** 2018-11-27

**Authors:** Maria Malm, Timo Vesikari, Vesna Blazevic

**Affiliations:** Faculty of Medicine and Life Sciences, Vaccine Research Center, University of Tampere, Tampere, Finland

**Keywords:** norovirus, CD8 T cell epitope, HLA-A2^*^0201, multifunctional T cells, pentamer, ELISPOT IFN-gamma, cellular immunity

## Abstract

Norovirus (NoV) causes a substantial global burden of acute gastroenteritis in all age groups and the development of NoV vaccine is a high priority. There are still gaps in understanding of protective NoV-specific immunity. Antibody mediated immune responses have been widely studied, but in contrast, the research on NoV-specific human T cell-mediated immunity is very limited. We have recently reported NoV capsid VP1-specific 18-mer peptide (^134^SPSQVTMFPHIIVDVRQL^151^) to induce strong CD8^+^ T cell immune responses in healthy adult donors. This work extends to identify the precise NoV T cell epitope and the restricting human leucocyte antigen (HLA). Pentamer technology was used to detect HLA-A^*^0201-restricted T cell-mediated responses to 10-mer peptide ^139^TMFPHIIVDV^148^ of four healthy adult blood donors. Immunogenicity of the 10-mer epitope was confirmed by ELISPOT IFN-γ and intracellular cytokine staining (ICS) on flow cytometry. A population of CD3^+^CD8^+^ T lymphocytes binding to HLA-A^*^0201/TMFPHIIVDV pentamers was identified in two HLA-A^*^0201-positive donors. Recognition of the 10-mer epitope by T cells resulted in a strong IFN-γ secretion as shown by ELISPOT assay. In addition, ICS confirmed that high proportion (31 and 59%) of the TMFPHIIVDV epitope-responsive CD3^+^CD8^+^ T cells in the two donors had multifunctional phenotype, simultaneously producing IFN-γ, IL-2 and TNF-α cytokines. In the present study novel human NoV HLA-A^*^0201-restricted minimal 10-mer epitope ^139^TMFPHIIVDV^148^ in the capsid VP1 was identified. The HLA-peptide pentamer staining of T cells from healthy donor PBMCs and cytokine responses in *ex-vivo* ELISPOT and ICS assays suggest that this epitope is recognized during NoV infection and activates memory phenotype of the epitope-specific multifunctional CD8^+^ T cells. The importance of this epitope in protection from NoV infection remains to be determined.

## Introduction

Noroviruses (NoV) are group of very contagious viruses that cause >90% of non-bacterial and approximately half of all-cause epidemic gastroenteritis worldwide. NoV gastroenteritis is a significant public health problem with high clinical and economic costs, that is estimated to cause over 200,000 deaths annually, mainly in resource-limited countries (1). Despite scientific efforts and increasing awareness of NoV burden, there is no vaccine available yet (2, 3). NoV exhibits a great genetic diversity with six currently recognized genogroups (GI-GVI), human NoVs belonging mainly to GI and GII, that include over 30 genotypes and numerous intra-genotype variants (4). GII.4 genotype has been predominant since the mid-90s, including pandemic variants US 1995/96, Farmington Hills 2002, Hunter 2004, New Orleans (NO) 2009, and most recently, Sydney 2012 (5). GII.4 variants share >95% of their VP1 capsid amino acid (aa) sequence, whereas < 85% of the capsid aa are identical between different genotypes (6). The antigenic diversity of NoVs is likely to have major impact on evading host immune responses and complicates designing of NoV vaccines. NoV research is further limited due to the lack of small animal model and efficient NoV propagation in cell culture (7, 8), but spontaneously formed NoV VP1 virus-like particles (VLPs) are successfully utilized as viral surrogates for assaying immune responses and as vaccine candidates (9, 10).

NoV infections cause acute but self-limited illness already at early age and several consecutive infections occur frequently (11–13). Protective immunity and clearance of NoV infections are not well-characterized (1, 2). Even though there is some indication of pre-existing antibodies conferring protection (14) the level of serum NoV-specific antibodies is not directly linked to protection from infection (15). The best correlate of protection from NoV infection identified so far are blocking (neutralizing) antibodies, that are able to block the binding of NoV VLPs to the putative receptors, histo-blood group antigens (HBGA's) (15). NoV-specific antibodies are cross-reactive to some extent within genogroups however, blocking antibodies are mostly genotype-specific with low protective capacity against more distinct strains (1). Although NoV-specific antibodies are extensively studied very little is published on human T cell immunity to NoV and its' role in protection (16–19). Studies using a murine norovirus (MNV)-mouse model have suggested T cell-mediated immunity to be important in clearance of MNV infection (20, 21). To this end, cytotoxic CD8^+^ T cell-mediated immune responses may provide substantial protection against serologically distinct viruses via recognition of cross-reactive, conserved epitopes, such as observed in influenza virus infection (22).

Using matrix peptide approach we have recently identified a NoV-specific 18-mer peptide (^134^SPSQVTMFPHIIVDVRQL^151^) containing CD8^+^ T cell epitope likely restricted to human leukocyte antigen (HLA)-A^*^0201 allele (17). Furthermore, peptide binding prediction tools have predicted 10 amino acid (aa) stretch as a minimal epitope (^139^TMFPHIIVDV^148^). In here, experiments were undertaken to experimentally confirm the recognition of the 10-mer epitope and its presentation by the HLA-A^*^0201 allele.

## Materials and methods

### Blood donors and cell isolation

Four healthy adults (age 35–45, laboratory personnel) volunteering in a study recently conducted by our laboratory were selected based on their CD8^+^ T cell responses to NoV GII.4 VP1-specific 18-mer peptide ^134^SPSQVTMFPHIIVDVRQL^151^ (originally named 99-20) recently described (17). The two peptide responders were HLA typed as HLA-A^*^02:01 carriers (17). The cells of the two donors identified as non-responders to the 18-mer peptide were used as negative controls. Peripheral blood mononuclear cells (PBMCs) of the heparin blood sample were obtained by Ficoll-Pague PLUS (GE Healthcare, Little Chalfont, United Kingdom) density gradient centrifugation. PBMCs were frozen in 10% DMSO in fetal bovine serum (FBS) using freezing container (Mr. Frosty™, Thermo Scientific,Waltham, MA, United States) with controlled rate of cooling at −80°C and transferred to liquid nitrogen. Prior to analysis, PBMCs were thawed, washed, and resuspended in culture medium (CM) containing RPMI 1640 with Glutamax® and HEPES (Gibco™ by Thermo Fisher Scientific) supplemented with 10 μg/ml Gentamicin (Gibco™) and 10% fetal bovine serum (FBS, Sigma-Aldrich, St. Louis, MO, United States). The samples tested here are collected at a single bleed. Each sample aliquot was tested simultaneously by pentamer staining, enzyme-linked immunosorbent spot interferon-gamma (ELISPOT IFN-γ) assay and intracellular cytokine staining (ICS) assays, at least two times. Written informed consent was obtained from each volunteer prior to the sample collection in accordance with the Declaration of Helsinki. No approval by an ethics committee was required as per the local legislation.

### Synthetic peptides and pentamers

To predict the optimal HLA-A^*^02:01 allele binding epitope within the 18-mer NoV-specific peptide ^134^SPSQVTMFPHIIVDVRQL^151^, artificial neural networks (ANN) (23) implemented at Immune Epitope Database and Analysis Resource (IEDB) was employed (24–26). A 10-mer high binding affinity sequence ^139^TMFPHIIVDV^148^ was identified (17) and the peptide was synthetized with purity >75% (Synpeptide Co. Ltd, Shanghai, China). SYFPEITHI prediction algorithm (http://www.syfpeithi.de) (27) was used for scoring the peptide affinity for HLA-A^*^0201 allele, as a binding score of >21 ensures synthesis of a custom pentamer by Proimmune Ltd. (Oxford, United Kingdom). HLA-A^*^0201/TMFPHIIVDV pentamer (Pro5® MHC Class I Pentamer) labeled with R-phycoerythrin (R-PE) was synthetized by Proimmune Ltd. In addition, HLA-A^*^0201 negative control pentamer conjugated to R-PE was synthetized as well. The degree of conservation of the 10-mer epitope among different NoV genotypes and genogroups was investigated using Basic Local Alignment Search Tool (BLAST) for sequence identity. Evolutionary analyses of the major capsid VP1 aa sequence of the aligned NoV genotypes were conducted in MEGA X (28). The evolutionary distances were computed using the Poisson correction method (29).

### Pentamer staining

PBMCs were treated for 10 min with Human BD Fc Block to prevent non-specific staining. To discriminate viable from non-viable cells, Horizon Fixable Viability stain 780 was used according to manufacturer's instructions. The cells (1 × 10^6^) were incubated for 30 min on ice with either HLA-A^*^0201/TMFPHIIVDV pentamer or negative control pentamer, using 0.25 or 0.5 μg of the pentamers per condition. Cells were further stained with monoclonal antibodies against human CD3 (clone UCHT1, fluorescein isothiocyanate (FITC) conjugate) and CD8a (clone RPA-T8, PerCP Cy5.5 conjugate) for 30 min on ice. All reagents used for staining were purchased from BD Pharmingen (San Jose, CA, United States). After washing the cells were resuspended in 1% FBS, 2.5% formaldehyde in PBS for flow cytometry acquisition. At least 400 000 events were acquired for analysis on a 2-laser FACS CantoII flow cytometer (BD) with FACSDiva Software V 6.1.3 (Becton Dickinson, Heidelberg, Germany). Data were analyzed by FlowJo software version 10.1 (Tree Star, San Carlos, CA, United States). Lymphocytes were gated according to forward/sideward scatter (FSC/SSC) and dead cells were excluded by gating on the population negative for the viability dye. CD3^+^CD8^+^ population was further plotted on SSC-A vs. pentamer population.

### ELISPOT IFN-γ assay

PBMCs of the four donors were assayed in an ELISPOT assay for IFN-γ production as previously described (17). The cells were stimulated with increasing concentrations of 10-mer ^139^TMFPHIIVDV^148^ peptide (0.05, 0.1, 0.5, 1, 2, and 4 μg/ml final concentration), 4 μg/ml of 18-mer peptide 99-20, 4 μg/ml irrelevant 9-mer peptide (negative control) or 50 μg/ml of phytohemagglutinin (PHA, positive control). Briefly, ninety-six-well nitrocellulose filter plates (Millipore) were coated with anti-human IFN-γ capture antibody (Mabtech) and blocked with 10% FBS in CM. PBMCs (0.2 × 10^6^ cells/well) were plated with stimulants and incubated for 20 h at +37°C and 5% CO_2_. Biotinylated anti-human IFN-γ antibody (Mabtech) followed by streptavidin-HRP (BD, New Jersey, United States) was used for detection. The spots were developed with Vector Nova Red substrate (Vector Labs, Burlingame, United States) and the plates were analyzed using ImmunoSpot Series II analyzer (CTL Europe, Leinfelden-Echterdingen, Germany). The results are expressed as mean spot forming cells (SFC)/10^6^ PBMCs of the duplicate wells.

### Intracellular cytokine staining (ICS)

An ICS assay was employed to quantify IFN-γ, TNF-α, and IL-2 producing CD3^+^ CD8^+^ T cells. PBMCs were stimulated according to the previously published protocol (17) with 4 μg/ml of 10-mer peptide ^139^TMFPHIIVDV^148^, 4 μg/ml irrelevant 9-mer peptide (negative control) or 1 μg/ml Staphylococcal enterotoxin B (SEB, Sigma) in the presence of 1 μg/ml CD28 and 1 μg/ml CD49d costimulatory antibodies (BD Biosciences, San Jose, CA, United States). PBMCs incubated in CM supplemented with the costimulatory antibodies only were used as additional control. The protein transport inhibitor brefeldin A (GolgiPlug, BD Biosciences, San Jose, CA, United States) at a concentration of 10 μg/ml was added after 2 h and the incubation was continued for 16 h at 37°C. After stimulation, the cells were treated with EDTA for 15 min and washed with FACS Stain buffer. Prior to fixation and permeabilization for ICS, PBMCs were blocked for non-specific staining, stained for viable/non-viable discrimination and surface markers CD3 and CD8a as described above for the pentamer staining. BD Fixation/Permeabilization solution was used according to the manufacturer's instructions and cells were intracellularly stained with the mixture of IFN-γ (clone 4S.B3) PE-Cy7 conjugate, IL-2 (clone MQ1-17H12) PE-conjugate, and TNF-α (clone MAb11) allophycocyanin (APC)-conjugate in 50 μl Perm/Wash buffer for 30 min on ice in the dark. Cells were resuspended in FACS Staining Buffer for acquisition and analysis using FACS CantoII flow cytometer and FACSDiva Software V 6.1.3. All reagents used for ICS were purchased from BD Pharmingen (San Jose, CA, United States). The data analysis was performed using FlowJo software version 10.1.

## Results

### The 10-mer minimal epitope prediction and conservation

**Figure 1:**
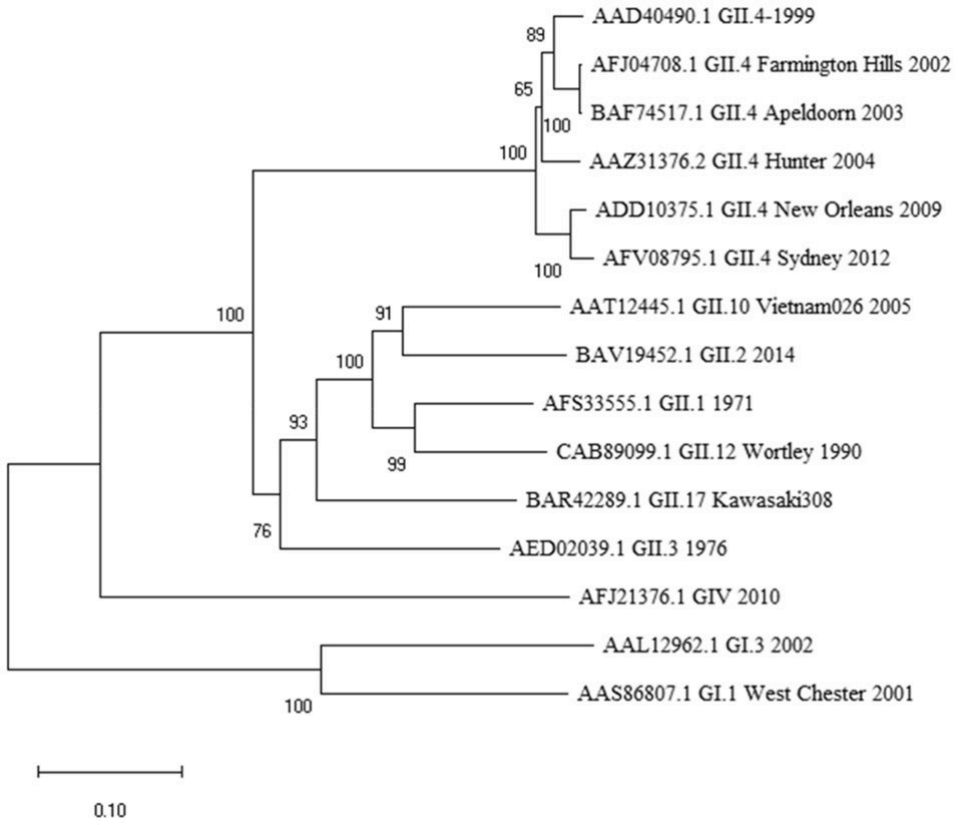
Phylogenetic analysis of norovirus major capsid protein VP1 amino acid sequences. The tree was inferred by Neighbor-Joining method with a bootstrap of 500 by using MegaX. The scale bar shows the genetic distance, expressed as amino acid substitutions per site.

At the outset of our study, an 18-mer NoV VP1-specific CD8^+^ T cell epitope (99-20, ^134^SPSQVTMFPHIIVDVRQL^151^) had been identified in two subjects, both having HLA-A^*^0201 allele. When analyzed by IEDB database ANN method, a 10 aa sequence ^139^TMFPHIIVDV^148^ within the 18-mer showed the highest binding affinity of IC_50_ 21.5 nM (Table 1), whereas all other predicted sequence lengths indicated affinity >80 nM (data not shown). Peptides with IC_50_ < 50 nM are considered high affinity and < 500 nM intermediate affinity (30). Congruently, the same 10-mer epitope was identified to have the highest binding affinity by SMM method [data not shown (23)]. SYFPEITHI prediction algorithm confirmed a score of 27 for the peptide affinity for HLA-A^*^0201 allele (Table 1). When comparing the aa sequence of different NoV genotypes (Table 1) the 10-mer epitope was found to be highly conserved among different GII.4 variants and also more distant NoV genotypes belonging to GII and GI. The phylogenetic distances of the NoV genotypes compared in the Table 1 are shown in Figure 1. In addition, the 10-mer epitope sequence in all genotypes belonging to GI and GII NoV listed in the Table 1 also had good affinity binding scores for HLA-A^*^0201 allele regardless of up to three aa substitutions compared to the GII.4-1999 10-mer sequence. On the contrary, the 10-mer sequence in the human GIV 2010 NoV had a unique substitution ^140^M to ^140^Q which seemed to abolish its binding affinity resulting in IC_50_ 552.6 nM and syfpeithi score 17 (Table 1). In addition, a change of the aa ^148^V to ^148^D at the c-terminus of the empirical 10-mer peptide abolished the predicted binding completely. This is not surprising as the aa at positions 140 and 148 are reported anchoring positions for HLA-A^*^0201 binding (31–33). In addition, deletions at the each terminus of the peptide affected the predicted binding capability of the peptide.

**Table 1:** Conservation and HLA-A2*0201 allele binding affinity prediction of the norovirus 10-mer epitope.

**Norovirus Genotype**	**Accession number**	**10-mer aa sequence (aa 139–148)**	**Identity %**	**IC_50_ nM (ANN)^a^**	**Syfpeithi score^b^**
GII.4-1999	AAD40490.1	T**M**FPHIIVD**V**	100	21.5	27
GII.4 Farmington Hills 2002	AFJ04708.1	TMFPHIIVDV	100	21.5	27
GII.4 Apeldoorn 2003	BAF74517.1	TMFPHIIVDV	100	21.5	27
GII.4 Hunter 2004	AAZ31376.2	TMFPHIIVDV	100	21.5	27
GII.4 New Orleans 2009	ADD10375.1	TMFPHIIVDV	100	21.5	27
GII.4 Sydney 2012	AFV08795.1	TMFPHIVVDV	90	28.22	27
GII.17 Kawasaki308	BAR42289.1	TMLPHLIVDV	80	30.14	28
GI.3 2002	AAL12962.1	TMFPHVIADV	80	14.44	27
GII.12 Wortley 1990	CAB89099.1	TMFPHVIIDV	80	31.84	25
GII.10 Vietnam026 2005	AAT12445.1	TMFPHVIIDV	80	31.84	25
GII.1 1971	AFS33555.1	TMFPHVIIDV	80	31.84	25
GII.2 2014	BAV19452.1	TMFPHVIIDV	80	31.84	25
GII.3 1976	AED02039.1	TMCPHVIVDV	80	278.56	26
GI.1 West Chester 2001	AAS86807.1	TLFPHVIADV	70	16.87	29
*Human GIV 2010*	*AFJ21376.1*	*TQFPHVIIDV*	*70*	*552.6*	*17*
*Not existing*		*TMFPHIIVDD*	*90*	*17260.44*	*17*
*9-mer*		*_MFPHIIVDV*	*90*	*4645.06*	*16*
*9-mer*		*TMFPHIIVD_*	*90*	*9837.8*	*15*

a *Inhibitory concentration (IC)_50_ values were obtained using IEDB analysis resource ANN aka NetMHC (ver. 4.0) tool*.

b Syfpeithi score was derived from the Epitope Prediction tool by http://www.syfpeithi.de/ Bolded letters indicate the reported anchor residues of HLA-A2*0201 allele binding epitopes (31, 32). Underlined letters indicate the aa changes with respect to GII.4-1999-derived 10-mer epitope. Italic font denotes sequences with no affinity binding to HLA-A2*0201.

### CD8^+^ T lymphocytes bind the 10-mer peptide ^139^TMFPHIIVDV^148^ in the context of HLA-A^*^0201

**Figure 2:**
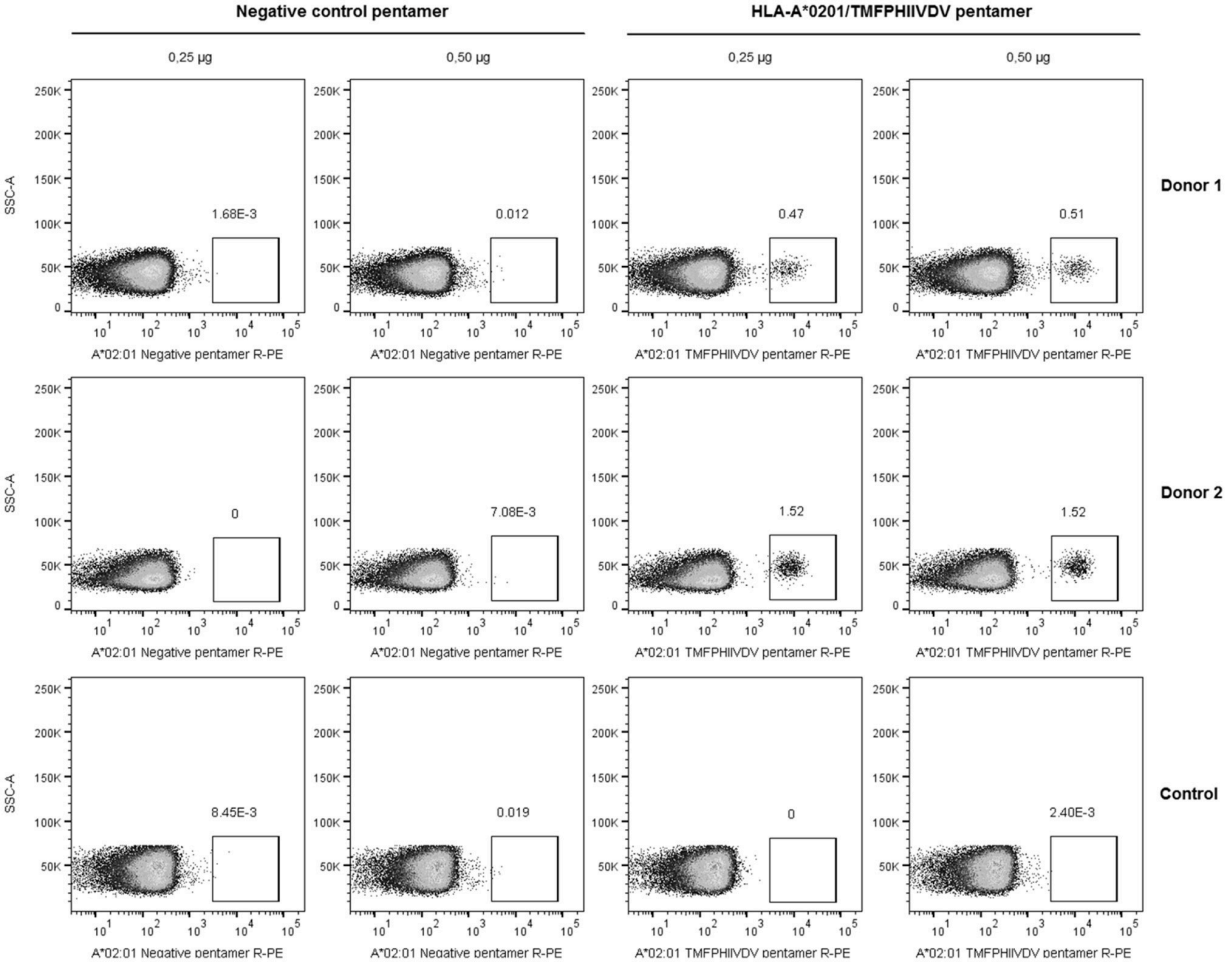
Pentamer staining of norovirus-derived ^139^TMFPHIIVDV^148^ epitope-specific CD8^+^ T cells. Peripheral blood mononuclear cells (PBMCs) from two HLA-A*0201-positive healthy adult donors and the negative control were directly stained using 0.25 and 0.5 μg HLA-A^*^0201/TMFPHIIVDV pentamer or negative control pentamer. The dot plots show the percentage of pentamer-positive T cells for two HLA-A*0201-positive donors and a representative negative control donor after gating on live CD3^+^CD8^+^ T lymphocyte population.

The frequency of T cells specific for ^139^TMFPHIIVDV^148^ 10-mer in the context of HLA-A^*^0201 allele was analyzed by direct binding to pentamer A^*^0201/TMFPHIIVDV R-PE using four-color flow cytometry. PBMCs of the two HLA-A^*^0201 positive donors previously responding to the 18-mer peptide 99-20 had frequency of 0.51% (Donor 1) and 1.52% (Donor 2) (Figure 2) pentamer-binding T cells of the live CD3^+^CD8^+^ gated lymphocytes. At the same time, the CD8^+^ T cells of the control donors non-responders to the 99-20, did not bind to A^*^0201/TMFPHIIVDV pentamer (Figure 2). The negative control pentamer did not show non-specific binding (Figure 2).

### The 10-mer ^139^TMFPHIIVDV^148^ epitope induces strong IFN-γ response in HLA-A^*^0201 positive donors

**Figure 3:**
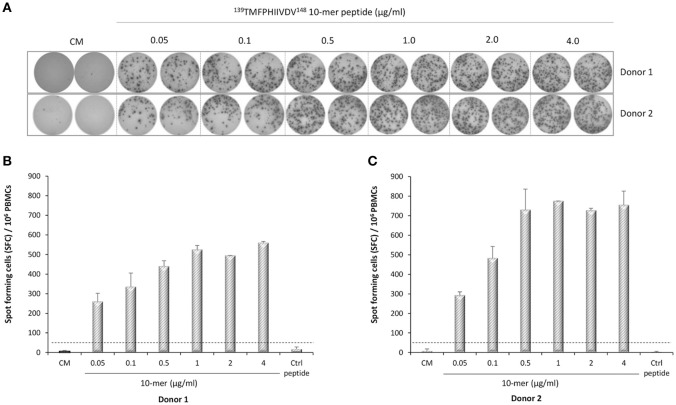
Norovirus epitope ^139^TMFPHIIVDV^148^ –specific enzyme-linked immunosorbent spot (ELISPOT) interferon-gamma (IFN-γ) responses. PBMCs of Donor 1 **(A,B)** and Donor 2 **(A,C)** positive for HLA-A^*^0201 allele were stimulated with an increasing concentration (0–4 μg/ml) of the 10-mer epitope peptide, 4 μg/ml irrelevant 9-mer peptide (Ctrl), or left unstimulated (CM, culture media). Images of the ELISPOT replicate wells with the stained spot-forming cells (SFC) are shown **(A)**. Mean SFC/10^6^ PBMCs of two replicate wells with the standard errors of the mean for Donor 1 **(B)** and Donor 2 **(C)**. The cut-off line (dotted line) indicates the positive SFC/10^6^ value of ≥50 SFC/10^6^ cells and twice above the background control (CM only wells).

IFN-γ-secreting lymphocytes specific for ^139^TMFPHIIVDV^148^ 10-mer peptide were enumerated by *ex*-*vivo* ELISPOT assay with increasing concentration of the 10-mer peptide. Robust IFN-γ response to the 10-mer peptide was observed for the two HLA-A^*^0201 positive donors (Figure 3) but not for the two control donors (data not shown). A dose response was observed up to 1.0 μg/ml concentration of the peptide and also very low concentration (0.05 μg/ml) stimulated high IFN-γ secretion (donor 1, 260 SFC/10^6^ cells; donor 2, 293 SFC/10^6^ cells). IFN-γ production was not observed in the cells stimulated with the irrelevant 9-mer peptide or CM only (SFC/10^6^ cells <50) (Figure 3A–C). All donors responded strongly to the positive control PHA (IFN-γ SFC/10^6^ cells >2000, data not shown).

### The 10-mer ^139^TMFPHIIVDV^148^ epitope induces multifunctional CD8^+^ T cells producing IFN-γ, IL-2, and TNF-α

**Figure 4:**
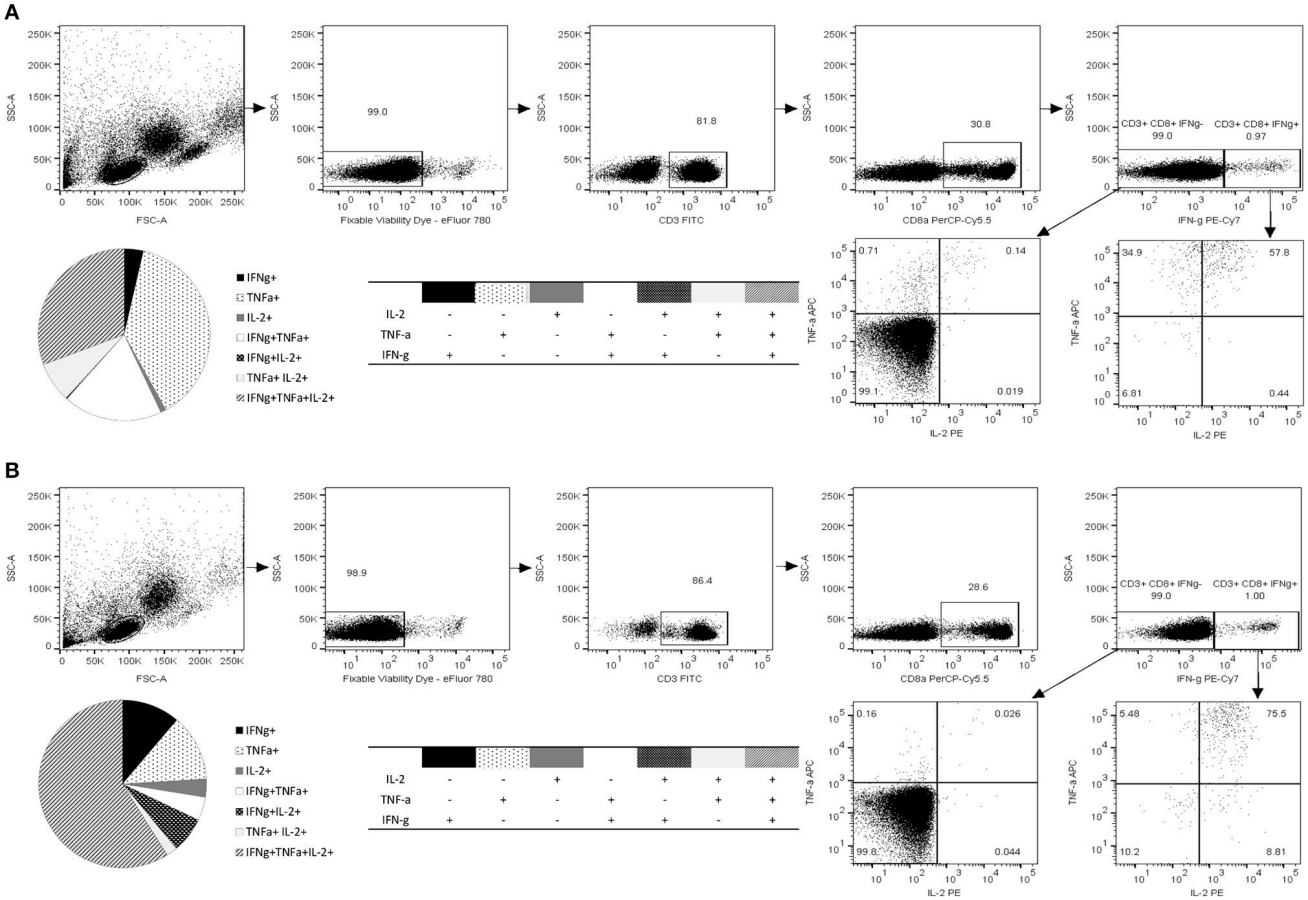
Functional characterization of norovirus 10-mer epitope ^139^TMFPHIIVDV^148^ –specific CD8^+^ T cells by intracellular cytokine staining (ICS). Production of IL-2, IFN-γ, and TNF-α measured by ICS after 16 h stimulation of the PBMC with the peptide ^139^TMFPHIIVDV^148^ (4 μg/ml). Shown are the results of the two HLA-A^*^0201-positive donors, Donor 1 **(A)** and Donor 2 **(B)**. Live epitope-specific CD3^+^ CD8^+^ T cells were segregated into IFN-γ^+^ and IFN-γ^−^ T cells. Seven distinct populations, based on production of the cytokines in any combination are depicted on the pie charts and the accompanying tables. Fractions of the cells producing one, two, or three cytokines are derived from the plotted IFN-γ^+^ and IFN-γ^−^ populations.

Along with quantification of the epitope-specific CD8^+^ T cells with HLA-A^*^0201 pentamer and IFN-γ ELISPOT, immune responses to 10-mer peptide were characterized by ICS of IFN-γ, IL-2, and TNF-α (Figure 4). The gating strategy for ICS analysis is shown in Figure 4. The viability of the lymphocytes was >98% and approximately 30% of the viable CD3^+^ lymphocyte population were CD8^+^ T cells. CD8^+^ T cells were further segregated into IFN-γ^+^ and IFN-γ^−^ population and plotted for the expression of IL-2 and TNF-α. Robust cytokine response by CD3^+^CD8^+^ T cells toward 10-mer epitope in donor 1 (Figure 4A) and donor 2 (Figure 4B) but not in two control donors (data not shown) was observed. Both donors had CD8^+^ T cells producing single, double, and triple cytokines (Figures 4A,**B**). App. 1% of the CD8^+^ T cells of both donors produced IFN-γ in response to the 10-mer epitope (Figure 4) whereas irrelevant control peptide induced no IFN-γ (< 0.05%, data not shown). The 10-mer epitope induced multifunctional CD8^+^ T cells in both donors, donor 1 had ~30% triple cytokine (IFN-γ^+^, TNF-α^+^, and IL-2^+^) secreting CD3^+^CD8^+^ T cells, while donor 2 had ~60% of the triple-positive CD8^+^ T cells. Strong IFN-γ, IL-2, and TNF-α response was observed in both CD8^+^ and CD8^−^ PBMCs against positive control SEB in all tested donors (not shown).

## Discussion

Despite significant efforts to define correlates of protection to NoV infection (1, 2), the role of T lymphocytes in protection and clearance of NoV infection is still largely unexplored area, with only few papers published so far (16–19, 34). We have recently tested ten healthy volunteer donors for NoV GII.4 capsid VP1-specific T cell responses using matrix peptide pools and found two CD8^+^ T cell responders to 18-mer NoV-specific peptide (^134^SPSQVTMFPHIIVDVRQL^151^, 99-20) (17). In here, we defined a 10-mer ^139^TMFPHIIVDV^148^ minimal epitope within this sequence and its restriction to HLA-A^*^0201 allele.

While the 10-mer ^139^TMFPHIIVDV^148^ epitope is derived from GII.4-1999 strain (17), the alignment with several other GII.4 variants showed epitope sequence to remain invariable until 2012 (Table 1). Interestingly, alignment with other quite distant genotypes (Figure 1) even belonging to GI viruses, indicated high degree of conservation of the epitope. Importantly, up to three aa substitutions as in the GI.1 West Chester 2001 NoV do not necessarily reduce the binding affinity to HLA-A^*^0201, depending on the aa position and characteristics. Aa at the position 6 and 8 of the 10-mer showed most variability among the aligned genotypes, however, these changes did not lead to considerable change in binding affinity predictions. However, a change at aa position 2 from a hydrophobic (M) to a polar aa (Q) in a human GIV 2010 NoV and at aa position 9 from V to D in an empirical sequence seemed to drastically abolish binding affinity. This is not surprising as HLA-A^*^0201 binding epitopes have a restricted size of 9/10 aa and hydrophobic anchor residues at positions 2 and 9/10 likewise the epitope identified in this study. The NoV 10-mer epitope contains methionine (M) at position 2 and valine (V) at position 10 which is described as a typical C-terminal anchor residue (31–33).

Direct staining of PBMCs with HLA-A^*^0201 pentamer loaded with ^139^TMFPHIIVDV^148^ peptide attested relatively high frequency of circulating NoV capsid-specific memory CD8^+^ T cells in two HLA-A^*^0201 positive healthy donors (0.51 and 1.52% of CD3^+^CD8^+^ gated lymphocytes, respectively). HLA-A2 is the most prevalent MHC allele family in human population and the gene frequency of HLA-A^*^02:01 is especially high in Caucasians (>20%), and other western ethnic groups (African Americans 12%, Hispanics 23%, North American natives 21%) (35–37), further emphasizing the importance of the identified epitope. However, it has been reported that only a small portion of the peptides with predicted and experimentally confirmed high affinity binding, are actually capable to induce T-cell responses (38). Therefore, the utilization of functional assays is essential for determining the immunogenicity of an epitope. To that end, the functionality of T cell immune responses to 10-mer epitope were determined by two functional assays, ELISPOT IFN-γ and ICS. The results of ELISPOT IFN-γ assay showed robust NoV VP1 10-mer epitope-specific CD8^+^ T cell immune responses in HLA-A^*^0201-positive individuals, whereas control donors were negative. Titration of 10-mer peptide down to 0.05 μg/ml revealed high avidity of CD8^+^ T cells to 10-mer peptide. These results were expanded using ICS assay to detect CD8^+^ T cells secreting cytokines IFN-γ, IL-2, and TNF–α. By this approach, the presence of circulating monofunctional and polyfunctional memory CD8^+^ T lymphocytes specific for 10-mer NoV epitope was detected in HLA-A^*^0201-positive healthy donors. Polyfunctional CD8^+^ T cells that produce more than one immune mediator, are associated with protection from viral infections, such as HIV-1 and human herpes virus (39–41). In the future we aim to utilize the pentamer technology to validate CD8^+^ T cell responses in a larger number of serologically positive NoV-infected HLA-A^*^02:01 positive subjects to strengthen the clinical significance of the finding in this study.

Pentamer staining, ELISPOT IFN-γ and ICS assays, performed simultaneously using the same sample of each donor, resulted in highly congruent data supporting high immunogenicity of the novel NoV ^139^TMFPHIIVDV^148^ CD8^+^ T cell epitope. Many viral infections are not contained by antibody responses alone and it is likely that T cell responses play a role in clearance of NoV infection and may have a role in protective immunity as well. Virus-specific CTL responses play a crucial role in a clearance of many other viral infections such as human immunodeficiency virus-1 (HIV-1) (40, 42, 43), influenza (22, 44) and human papilloma virus (45). Patients with chronic hepatitis B (HBV) infection typically lack effective HBV-specific T cells, whereas fully recovered patients display strong CD8^+^ T-cell responses (46).

To conclude, the present work describes the first human NoV CD8^+^ T cell epitope ^139^TMFPHIIVDV^148^ restricted to HLA-A^*^02:01 allele. Regarding the high conservation of the identified epitope and high frequency of HLA-A^*^02:01 allele in human population, it can be speculated that ~20% of the individuals exposed to divergent NoV genotypes, will develop strong CD8^+^ T cell responses to this 10-mer epitope. While NoV blocking antibodies are largely genotype specific, a role of T cells targeted to broadly conserved epitopes, as described in here, may be of large significance. However, the role and importance of this epitope as well as overall T cell responses in protection from NoV infection needs to be further investigated.

## Author contributions

MM sample acquisition, processing, and laboratory analysis (Pentamer staining, ELISPOT, and ICS by flow cytometry). Data acquisition, analysis, and interpretation, and writing the manuscript. TV the Head of Vaccine Research Center, revision of the manuscript text. VB the Head of the Laboratory, conception and designing the study, data interpretation, drafting, and writing the paper, critical revision of article for important intellectual content.

### Conflict of interest statement

The authors declare that the research was conducted in the absence of any commercial or financial relationships that could be construed as a potential conflict of interest.
